# Intra- and postoperative adverse events in awake craniotomy for intrinsic supratentorial brain tumors

**DOI:** 10.1007/s10072-020-04683-0

**Published:** 2020-08-17

**Authors:** Borys M. Kwinta, Aneta M. Myszka, Monika M. Bigaj, Roger M. Krzyżewski, Anna Starowicz-Filip

**Affiliations:** 1 Department of Neurosurgery and Neurotraumatology, Jagiellonian University Medical College, Jakubowskiego 2 Street, 30-688 Kraków, Poland; 2 Jagiellonian University Medical College, Kraków, Poland; 3 Department of Anesthesiology, 5th Military Hospital in Krakow, Krakow, Poland

**Keywords:** Awake craniotomy, Intrinsic brain tumor, Adverse events, Complications, Seizures, Hypertension

## Abstract

**Objective:**

To determine the frequency and consequences of intra- and postoperative adverse events in awake craniotomy for intrinsic supratentorial brain tumors. Despite the growing prevalence of awake craniotomy intra- and postoperative, adverse events related to this surgery are poorly discussed.

**Methods:**

We studied 25 patients undergoing awake craniotomy with maximum safe resection of intrinsic supratentorial brain tumors in the awake-asleep-awake protocol.

**Results:**

Surgery-related inconveniences occurred in 23 patients (92%), while postoperative adverse events were observed in 17 cases (68%). Seven patients suffered from more than one postoperative complication. The most common surgery-related inconvenience was intraoperative hypertension (8 cases, 32%), followed by discomfort (7 cases, 28%), pain during surgery (5 cases, 20%), and tachycardia (3 cases, 12%). The most common postoperative adverse event was a new language deficit that occurred in 10 cases (40%) and remained permanent in one case (4%). Motor deficits occurred in 36% of cases and were permanent in one case (1%). Seizures were observed in 4 cases (16%) intra- and in 2 cases (8%) postoperatively. Seizures appeared more frequently in patients with multilobar insular-involving gliomas and in patients without prophylactic antiepileptic drug therapy.

**Conclusions:**

Surgery-related inconveniences and postoperative adverse events occur in most awake craniotomies. The most common intraoperative adverse event is hypertension, pain, and tachycardia. The most frequent postoperative adverse events are new language deficits and new motor deficits.

## Introduction

Awake craniotomy (AC) with cortical and subcortical brain mapping has become a standard method in the treatment of intrinsic brain tumors located in eloquent brain areas [1]. There is a growing body of evidence that awake craniotomy is an effective method for treatment of gliomas promoting a higher extent of resection, a lower incidence of neurological complications, and ensuring better overall survival while dealing with language and motor locations [2, 3]. Awake surgery is gaining attention in the field of other intracranial pathologies such as aneurysm repair, arteriovenous malformation excisions [4], and metastasis surgery [5]. When compared with surgery under general anesthesia, awake craniotomy for eloquent brain tumors ensures better onco-functional balance [6].

Despite wide discussion on the effectiveness of the awake craniotomy procedure, related complications are often neglected. Usually authors limit the presentation of complications to a superficial description of the outcome and basic deficits that occurred [7]. Therefore there is a need for identification of intra- and postoperative complications of awake surgery. The intra and postoperative complications may be related to surgical and anesthesiological factors. The most common surgical complications are: seizures and new neurological deficits. Complications related to anesthesia care like desaturation often lead to AC failure with a need for conversion to general anesthesia [8].

In the present study, we sought to determine the most common surgery and anesthesia care–related inconveniences and postoperative adverse events in patients undergoing AC for intrinsic brain tumors in eloquent brain locations and establish their frequency.

## Materials and Methods

We retrospectively analyzed the data of 25 patients who underwent maximum safe resection for intrinsic supratentorial brain tumors in an asleep-awake-asleep (SAS) setting between 2014 and 2017 in one neurosurgical center. The indication for awake craniotomy was a tumor located in an eloquent brain area. Patients with a substantial neurological deficit prior to surgery were not qualified for awake craniotomy. Substantial deficit was understood as a lack of ability to cooperate during the awake procedure or motor weakness greater than 3/5 according to the Lovett scale. We studied the detailed medical history, including chronic diseases and medication taken. All patients were assessed according to the American Society of Anaesthesiologists (ASA) Physical Status Classification System. All patients underwent neuropsychological evaluation and testing pre-, intra-, and postoperatively.

The asleep phase was achieved using the total intravenous anesthesia protocol (propofol and remifentanil) and the airways were secured with a laryngeal mask (i-gel®). The monitoring of the following parameters was performed throughout the procedure: mean arterial blood pressure, heart rate, end-tidal CO_2_ concentration (during the asleep phase, when the laryngeal mask was inserted), percentage of oxygenated hemoglobin, and temperature.

All patients were treated with a tailored craniotomy, large enough to perform positive brain mapping followed by the maximum safe resection of the tumor during the awake phase. Positive brain mapping was defined as cortical or subcortical current stimulation which provokes a temporary onset of neurological symptoms in awake patients, like speech arrest or motor weakness. The maximum safe resection was defined as the maximal possible tumor resection without inducing new neurological deficit and while controlled by intraoperative brain mapping techniques. Intraoperative seizures were managed with ice cold Ringer’s lactate. After tumor resection, during the wound closing procedure, mild sedation with remifentanil and propofol were used as well as pain control medications.

Surgery and anesthesia care–related inconveniences and postoperative adverse events were analyzed. Hypertension was defined as systolic blood pressure over 140 mmHg and diastolic over 90 mmHg. Hypotension was defined as systolic blood pressure under 90 mmHg and diastolic under 60 mmHg. Tachycardia was recognized when the heart rate exceeded 100 beats per minute, bradycardia when the heart rate was below 60 beats per minute. Desaturation was defined as SpO_2_ < 90%. Brain edema was reported by the operating surgeon if the brain exceeded the outer table of the craniotomy window. Nausea was recorded as reported by patients. Seizures and vomiting were recorded if they occurred during surgery. New neurological deficits (language or motor) were considered a new finding if present during the neurological examination directly after surgery. A transient deficit was recognized if this subsided within 30 days of surgery. A neurological deficit was defined as permanent if it was present after a 30-day follow-up. The postoperative period was defined as the first 30 days after surgery. The extent of resection based on control MR imaging was analyzed. All patients received an MRI three to six months postoperatively. The extent of resection was evaluated based on fluid-attenuated inversion recovery MRI sections.

Written consent was obtained from all patients involved into the study upon admission to the hospital. This study was conducted in accordance with the ethical standards laid down in the Declaration of Helsinki (1964) and its design was approved by the local University Bioethical Committee (protocol number 1072.6120.305.2018).

We used elements of descriptive statistics including mean, standard deviation, and percentage value to analyze our data.

## Results

The study group consisted of 25 patients (8 female, 32%). Mean age was 47.96 ± 15.97 years. The patients’ preoperative ASA scores were no worse than 3. The patients’ as well as their tumors’ characteristics are shown in Table 1. Among the patients, 17 diffused astrocytomas (68%), 4 anaplastic astrocytomas (16%), and 4 glioblastomas (16%) were diagnosed. Gross total resection was performed in 20 cases (80%). In 5 cases (20%), subtotal resection was achieved. All cases with a tumor diameter ≥ 5 cm preoperatively received subtotal resection.

**Table 1:** Patients’ characteristics including location and histopathology of gliomas

Characteristic	Patients	%
Age ± standard deviation	47.96 ± 15.87	
History of the patients		
Headache	21	84
First, single epileptic attack	12	48
More than one epileptic seizure	6	24
Numbness	8	32
Motor deficit	4	16
Smoking	6	24
Regular alcohol intake	1	4
Obesity	4	16
Arterial hypertension	8	32
Atherosclerosis	5	20
Hypercholesterolemia	1	4
Beta-blockers	2	8
Anticonvulsants	10	40
ACEI	2	8
Calcium channel blockers	1	4
Diuretics	5	20
Statins	1	4
Steroids	8	32
ASA I class	12	48
ASA II class	11	44
ASA III class	2	8
Tumor grade		
WHO II	17	68
WHO III	4	16
WHO IV	4	16
Location in the left hemisphere		
Frontal lobe	6	24
Temporal lobe	7	28
Parietal lobe	1	4
Multilobar insula involving	10	40
Tumor size		
≥ 5 cm	19	76
< 5 cm	6	24

Surgery-related inconveniences and postoperative adverse events occurred in 23 cases (92%) and are presented in Table 2. The most frequent intraoperative event was hypertension, which occurred in 8 cases (32%). Pain was present in 5 cases (20%) and tachycardia occurred in 3 cases (12%).

**Table 2:** Intra- and postoperative adverse events

Intraoperative adverse event	Number of patients	%
Arterial hypertension	8	32
Discomfort during surgery	7	28
Pain during surgery	5	20
Intraoperative seizures	4	16
Tachycardia	3	12
Psychological stress	3	12
Arterial hypotension	2	8
Bradycardia	1	4
Desaturation	1	4
Brain swelling	1	4
Nausea/vomiting	1	4
Aborted-converted to sleep	1	4
Postoperative adverse event		
New language deficit	10	40
New motor deficit	9	36
Postoperative seizures	2	8
Postoperative nausea/vomiting	2	8
Decreased conscious level	1	4
Permanent language deficit	1	4
Permanent motor deficit	1	4

Postoperative adverse events were noticed in 17 cases (68%). All patients suffered from more than one adverse event. Hypertension was present in five cases during the awake phase. All cases with pain or psychological stress coexisted with hypertension. Two cases of tachycardia were linked with pain, one with psychological stress. New language deficit was the most frequent postoperative adverse event and occurred in 10 cases (40%); it turned out to be permanent in one case (4%). The second most frequent was new motor deficit, which occurred in 9 cases (36%) and was permanent in one case (4%). Seizures were observed in 4 cases (16%) intra- and in 2 cases (8%) postoperatively. Seizures appeared more frequently in patients with multilobar insular-involving gliomas (4 patients, 16% of the study group and 66.66% of cases with treatment related seizures) and in patients without prophylactic antiepileptic drugs (5 patients, 20% of the study group and 83.33% of cases with treatment related seizures). One patient (4%) with an intraoperative seizure presented with desaturation and this resulted in eventual conversion to general anesthesia. All cases that presented with intraoperative seizures reported seizures prior to surgery. The incidence and occurrence of seizures are presented in Table 3. One patient, after a large multilobar tumor (diameter over 5 cm) resection, required decompressive craniectomy. The patient deteriorated within the first postoperative day (seizures, decreased consciousness level, hemiparesis). Control head CT revealed a brain edema and hemorrhagic progression. After emergency reoperation the patient recovered completely during the postoperative period. All seizure episodes as well as hemorrhagic complications occurred after large tumor (diameter over 5 cm) removal. Neither infections nor wound healing problems were observed in the study group.

**Table 3:** Incidence and occurrence of seizures

Evidence and type of seizures preoperatively	Number of the patients (%)	Prophylactic antiepileptic treatment (%)	Seizures intraoperatively (%)	Seizures postoperatively (%)	Multilobar insula involving gliomas (%)
No seizures	7 (28)	2 (8)	0	0	0
First, single epileptic attack	12 (48)	2 (8)	1 (4)	0	0
More than one epileptic seizure	6 (24)	6 (24%)	3 (12)	2 (8)	4 (16)

## Discussion

We analyzed the adverse events observed during and after awake craniotomy for intrinsic brain tumors according to our own experience.

We found the most frequent intraprocedural adverse event was hypertension. The adverse events registered most commonly after surgery were new language and motor deficits. More than 90% of the new deficits subsided within the postoperative period. Hypertension is rarely reported in awake craniotomy. Eseonu et al. reported hypertension being the most common complication of awake surgery [9] but limited data regarding its causality are available. Hypertension during awake surgery corresponds with pain or psychological stress and their overlapping. This thesis may be supported by the higher incidence of intraoperative hypertension in patients undergoing surgery under conscious sedation when compared with asleep-awake-asleep surgery [10]. In our setting, all patients underwent the asleep-awake procedure, to reduce potential stress related to the craniotomy procedure. Higher comfort and pain reduction are reported by other studies to be advantages of the asleep-awake-asleep procedure [10, 11].

Seizures leading in some cases to transient neurological deterioration were potentially serious complications. The use of prophylactic antiepileptic drugs for brain tumors in patients undergoing craniotomy remains controversial [12, 13], even in patients operated in an awake setting [14–16].

## Conclusions

With this paper, we would like to stress the need for analysis and prevention of adverse events during and after awake surgery for brain tumors. The need for reduction in the patient’s stress and pain is the first conclusion which can be drawn from our results. As seizures are a typical adverse event for gliomas and can be easily induced by brain stimulation intraoperatively or brain irritation after surgery, the use of prophylactic anticonvulsants seems to be the proper option while dealing with a patient treated with awake craniotomy.

The removal of large tumors is more prone to produce intraoperative seizures and these patients are at a higher risk of postoperative hemorrhage, according to our observation.
